# Integrity and Collaboration in Dynamic Sensor Networks

**DOI:** 10.3390/s18072400

**Published:** 2018-07-23

**Authors:** Steffen Schön, Claus Brenner, Hamza Alkhatib, Max Coenen, Hani Dbouk, Nicolas Garcia-Fernandez, Colin Fischer, Christian Heipke, Katja Lohmann, Ingo Neumann, Uyen Nguyen, Jens-André Paffenholz, Torben Peters, Franz Rottensteiner, Julia Schachtschneider, Monika Sester, Ligang Sun, Sören Vogel, Raphael Voges, Bernardo Wagner

**Affiliations:** 1 Institut für Erdmessung, Leibniz Universität Hannover, 30167 Hannover, Germany; dbouk@ife.uni-hannover.de (H.D.); garcia@mbox.ife.uni-hannover.de (N.G.-F.); lohmann@ife.uni-hannover.de (K.L.); 2 Institute of Cartography and Geoinformatics, Leibniz Universität Hannover, 30167 Hannover, Germany; Claus.Brenner@ikg.uni-hannover.de (C.B.); colin.kuntzsch@ikg.uni-hannover.de (C.F.); torben.peters@ikg.uni-hannover.de (T.P.); Julia.Schachtschneider@ikg.uni-hannover.de (J.S.); Monika.Sester@ikg.uni-hannover.de (M.S.); 3 Geodetic Institute, Leibniz Universität Hannover, 30167 Hannover, Germany; alkhatib@gih.uni-hannover.de (H.A.); Neumann@gih.uni-hannover.de (I.N.); paffenholz@gih.uni-hannover.de (J.-A.P.); ligang.sun@gih.uni-hannover.de (L.S.); vogel@gih.uni-hannover.de (S.V.); 4 Institute of Photogrammetry and GeoInformation, Leibniz Universität Hannover, 30167 Hannover, Germany; coenen@ipi.uni-hannover.de (M.C.); heipke@ipi.uni-hannover.de (C.H.); nguyen@ipi.uni-hannover.de (U.N.); rottensteiner@ipi.uni-hannover.de (F.R.); 5 Real Time Systems Group, Institute for Systems Engineering, Leibniz Universität Hannover, 30167 Hannover, Germany; voges@rts.uni-hannover.de (R.V.); wagner@rts.uni-hannover.de (B.W.)

**Keywords:** integrity, collaboration, GNSS, laser scanner, camera, interval mathematics, set theory

## Abstract

Global Navigation Satellite Systems (GNSS) deliver absolute position and velocity, as well as time information (P, V, T). However, in urban areas, the GNSS navigation performance is restricted due to signal obstructions and multipath. This is especially true for applications dealing with highly automatic or even autonomous driving. Subsequently, multi-sensor platforms including laser scanners and cameras, as well as map data are used to enhance the navigation performance, namely in accuracy, integrity, continuity and availability. Although well-established procedures for integrity monitoring exist for aircraft navigation, for sensors and fusion algorithms used in automotive navigation, these concepts are still lacking. The research training group i.c.sens, integrity and collaboration in dynamic sensor networks, aims to fill this gap and to contribute to relevant topics. This includes the definition of alternative integrity concepts for space and time based on set theory and interval mathematics, establishing new types of maps that report on the trustworthiness of the represented information, as well as taking advantage of collaboration by improved filters incorporating person and object tracking. In this paper, we describe our approach and summarize the preliminary results.

## 1. Introduction

The requirements for the navigational information (position, velocity, attitude and time) described in terms of accuracy, integrity, continuity and availability have grown considerably in recent years in order to ensure safe navigation. One of the key features for future autonomous systems is a guarantee of integrity, i.e., to warn the user in a timely manner when a predefined threshold (alert limit) is transgressed [1]. So far, integrity concepts have been developed and used primarily in aviation for GPS-based navigation [2,3]); an overview of studies for integrity in urban areas is given in [4]. One established solution to evaluate the integrity risk is Receiver Autonomous Integrity Monitoring (RAIM); cf. [5,6]. Over the past 25 years, many different RAIM approaches have been developed, e.g., [7,8]. Alternatively, diversified observation configurations or sensor systems are considered, which independently enable a state estimation. By integrating map information, another partially independent source of information can be used to increase integrity [9,10], but generally, the map itself has nowadays no dedicated integrity information.

Common to all integrity monitoring concepts is the need for a sound description of the contributing observation and system errors (which is similar to the approach of [11]), an over-determination of the navigational states and a good geometric configuration of the observations, which determines whether the data to be eliminated can be correctly detected and excluded [12,13]. However, the uncertainty modeling is done almost exclusively in a stochastic manner. This may not be adequate since the considered systems are too complex or dominant deterministic sources of uncertainty are observed [14,15].

In our work, we try to overcome these deficits by integrating integrity measures into universally-usable digital maps and develop alternative (interval based) integrity approaches. We rely on interval-mathematical descriptions (e.g., [16,17]), fuzzification [18,19] and combinations of probabilistic, interval-mathematical and fuzzy approaches [20,21,22]. Applications to vehicle navigation with GNSS have been reported in [23,24] and in subsequent work, guaranteed position estimation of robots in, e.g., [25,26,27,28].

In this paper, preliminary results are shown covering the topics of comparing different error bounding concepts in order to enclose the estimated positional states, adding interval bounds on time synchronization in multi-sensor platforms and including additional constraints in the filter solution. Integrity information for maps is explored by long-term repeated laser scans of an urban area in order to distinguish between varying and stable, thus trust-worthy, environments.

Moreover, the navigation quality can be improved by including several multi-sensor systems (equipped with GNSS, laser scanners, stereo cameras, Micro-Electro-Mechanical Systems (MEMS), odometers or radars) able to share information with each other under the same navigation scenario, in this way describing a dynamic collaborative network; cf. [29,30,31,32]. Thanks to local communication and computation capabilities, such sensor networks are not just a collection of individual sensors, but are capable of distributed computations, which leads to reliability and scalability [33].

**Figure 1:**
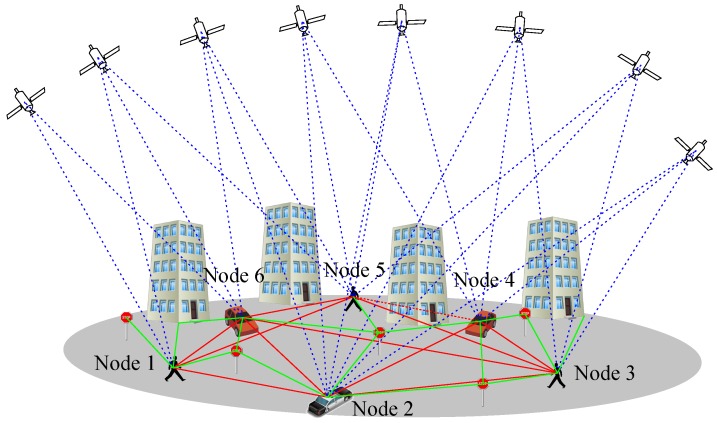
Collaborative positioning by vehicle-to-vehicle, vehicle-to-infrastructure and vehicle-to-pedestrian schemes. In addition, GNSS signals are received. The inter-nodal measurements are carried out using, e.g., laser scanners, stereo cameras and relative GNSS. Vehicle-to-infrastructure measurements are based on laser scanners and stereo cameras with dedicated processing and storage.

In the extension of Vehicle-to-X (V2X) communication schemes in collaborative environments [34], also relative measurement scenarios can be distinguished, namely Vehicle-to-Vehicle (V2V), Vehicle-to-Infrastructure (V2I) and Vehicle-to-Pedestrian (V2P); see Figure 1.

The cooperation takes place via the exchange of local maps on the basis of common features or by introducing relative observations; however, in most cases, the estimation itself is carried out under simplified conditions, e.g., in offices [35], or with the help of signalized targets on the robots [36,37]. With respect to the aggregation level of the observations, most works combine known relative poses of the different sensors [35,36,38,39]; for approaches involving the detection and localization of vehicles in images, see [40,41,42,43]. Specific feature extraction and storage have been developed [44,45,46]. Currently, maps are also cooperatively created in the area of Volunteered Geographic Information (VGI, e.g., OpenStreetMap), whereby users cooperatively monitor and ensure the integrity and consistency of the collected data.

In our perspective, the aim of collaboration is to improve the quality and to enable the positioning and navigation of individual users with the help of other users; this includes tracking of persons and other vehicles. To optimally solve a problem in the network, a special configuration and quality of sensors is required. For positioning in static networks, these questions were examined in the geodetic network optimization literature [47,48,49,50]. A dynamic change of the network topology has not been considered so far.

The overall developed methods will be applied to, tested and validated with dedicated measurements carried out by our research training group. Various scenarios in different urban environments in Hannover, Germany, were designed to allow collaborative positioning and navigation of multiple vehicles, as well as an evaluation of integrity. The obtained datasets and their storage are described in detail below.

The remainder of the paper is structured as follows. Section 2 gives an overview of the measurement campaigns (mapathons), as well as the concepts developed to ensure an adequate multi-user storage. Section 3 highlights intermediate results from the different individual research projects contributing to the topical area of integrity monitoring and collaborative navigation. Section 4 discusses the results achieved so far and presents conclusions.

## 2. Designing and Evaluating Dedicated Test Campaigns (Mapathons)

For highly automatic or autonomous driving applications, methods are required that enhance positioning accuracy and ensure integrity. Such methods are being developed for dynamic networks in the scope of the research training group, based on the fusion of multi-sensor and map data and on collaboration between network nodes. For a realistic implementation and evaluation of the developed approaches, a common spatial and temporal test environment is needed; see similar approaches discussed, e.g., in [51] or [52].

To generate the related data, we designed a comprehensive measurement campaign, which we refer to as a mapathon. More specifically, we created a dynamic sensor network consisting of three vehicles (vans of Geodetic Institute Hannover (GIH), Institut für Erdmessung (IfE) and Institute for Cartography and Geoinformatics (IKG)). They were equipped with multi-sensor platforms, simultaneously driving through selected urban areas located in Hannover, Germany. The sensors used are stereo-cameras, laser scanners, GNSS antennas and receivers, as well as Inertial Measurement Units (IMUs). A more detailed description of the sensor platforms is provided in the following Section 2.1. Further, we gathered data designing different real-time constellations of sensor nodes, realizing mutual vehicle observability and global time synchronization. A more detailed description of the mapped scenarios is given in Section 2.2.

### 2.1. Setups

To gather data, we equipped the three vans with different sensors, which will be explained in this section. On the one hand, each vehicle serves a distinct purpose or research question, which is of relevance to our research training group. On the other hand, the combination of all three cars allows us to explore the benefits of collaboration.

**Figure 2:**
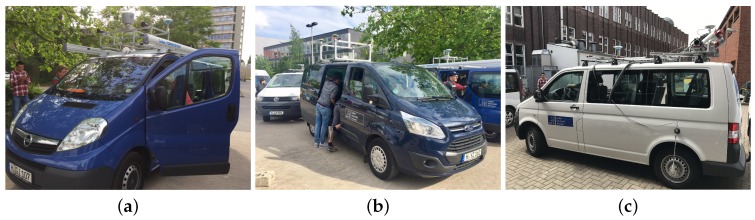
The participating vans with mounted sensor platforms during the mapathon: (**a**) GIH van, (**b**) IfE van and (**c**) IKG van.

The GIH van (Figure 2a) was equipped with a JAVAD Delta-G3T GNSS receiver (10 Hz) connected to a NavExperience antenna, a LORD MicroStrain 3DM-GQ4-45 GNSS aided IMU, a Velodyne HDL-64E laser scanner (10 Hz, 64 laser beams, 0.09° angular resolution), as well as a pair of stereo cameras (Camera: Pointgrey GS3-U3-23S6C-C, Lens: Tamron M111FM08; cf. Table 1). The Velodyne laser scanner, the Microstrain IMU and the stereo cameras were connected to an Ubuntu operating system via ROS nodes. A Windows operating system was used to control the JAVAD receiver. The sensors set up in this van are widely applied in modern highly automated cars.

The IfE van (Figure 2b) was equipped with an array of four JAVAD antennas connected to a JAVAD QUA-G3D Sigma receiver (10 Hz) and a NovAtel SPANE-SE receiver, which includes an iMAR FSAS IMU (200 Hz), and a Grashopper 2.3 stereo camera system. In addition to the position, velocity and time solution from the GNSS receivers, the vehicle orientation (attitude) can be derived from the antenna array and compared to (or combined with) the IMU measurements. Moreover, a tightly-coupled combined solution of antenna array and IMU can be used as a reference solution.

**Figure 3:**
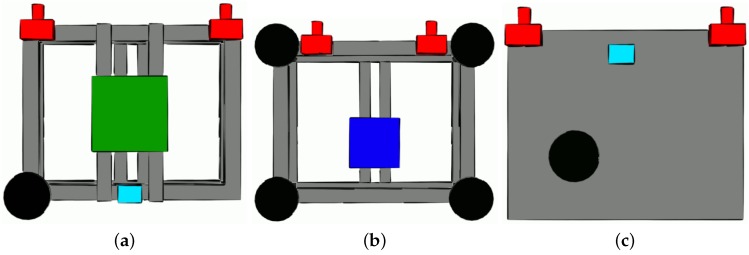
Sketches of the different multi-sensor platforms (top view) for the GIH van (**a**), IfE van (**b**) and IKG van (**c**), with their respective sensors (black: GNSS antenna; cyan: tactical-grade IMU; green: laser scanner; blue: tactical-grade IMU; red: camera).

The IKG van (Figure 2c) was equipped with a Riegl VMX-250 Mobile Mapping System, containing two laser scanners, a camera system and a localization unit. The localization is provided by a highly accurate GNSS/IMU system combined with an external distance measurement instrument (Applanix POS LV 510). The resulting trajectory typically has an accuracy of about 10–30 cm in height and 20 cm in position in urban areas. Each scanner has a measuring range up to 200 m with an accuracy of 10 mm. Additionally, this vehicle was also equipped with our own platform, consisting of a stereo camera pair and a GNSS system. The used stereo cameras Allied Vison AV MAKO G-234C have a base length of 0.85 m and point in the driving direction. The JAVAD TRE-G3TH delta (100 Hz) disciplined with a Chip Scaled Atomic Clock Microsemi (CSAC, [53]) was connected to a NavExperience antenna and a LORD MicroStrain 3DM-GQ4-45 GNSS-aided IMU. While the mobile mapping system is mainly used to collect data for projects related to digital map update, our own platform together with other vehicles was employed to acquire data to answer questions related to collaborative positioning and object tracking. The sensor configurations at the different platforms are depicted in Figure 3.

#### Calibration and Time Synchronization

**Figure 4:**
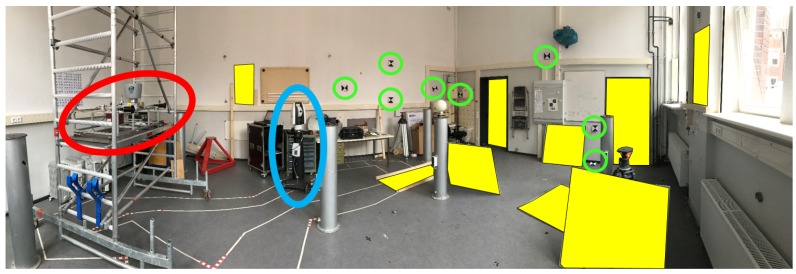
Multi-sensor calibration of one platform (red ellipse) by a laser tracker (blue ellipse) with control points for the stereo cameras (green circles) and planes for laser scanner (yellow surfaces), carried out in the 3D laboratory of the Geodetic Institute Hannover.

To combine all individual sensors into one joint multi-sensor system, the relative position and orientation of each sensor has to be known with respect to a fixed platform coordinate system. A laser tracker (Leica Absolute Tracker AT960) was used for defining such a temporally-stable coordinate system to ensure the highest precision requirements of less than 1 mm. Reference points of the IMUs and GNSS antennas were considered by exterior housing and construction drawings or mounting points. In contrast, 6 DOF (three translations and three rotations) information of the laser scanner and the stereo cameras were estimated indirectly using known reference planes and control points. To check for potential temporal changes during data acquisition, independent calibrations were carried out directly before and after the mapathon measurements; we determined differences in position with an average of 0.2±0.3 mm. Figure 4 shows the basic calibration setup, carried out in our 3D calibration laboratory. In addition, the platform coordinate system was linked to the vehicle body frames by measuring distinct points at the vehicles.

In order to time-synchronize the gathered sensor data, three u-blox EVK-M8T GNSS receivers were used. Each u-blox receiver was placed in one van. Because the Riegl mobile mapping system and the GPS of the IfE van provide their own time signal, they were not connected to the u-blox receivers. The received GPS time was fed via the GPS daemon (gpsd) to the network time protocol daemon (ntpd) of the attached Linux systems. The ntpd provides the timestamp for every sensor connected to the computer. We used UNIX time with a nanosecond resolution as our common time system.

### 2.2. Description of the Scenarios

**Figure 5:**
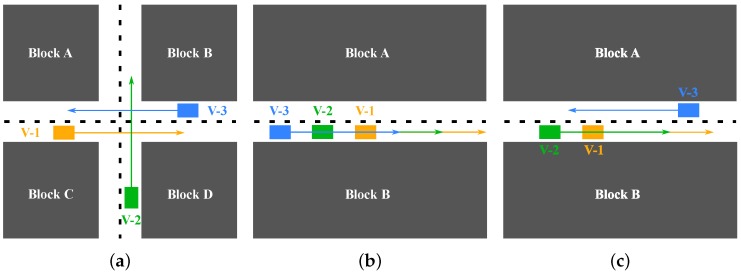
Sketch of three different collaborative scenarios (**a**–**c**), carried out during the Mapathon.

**Figure 6:**
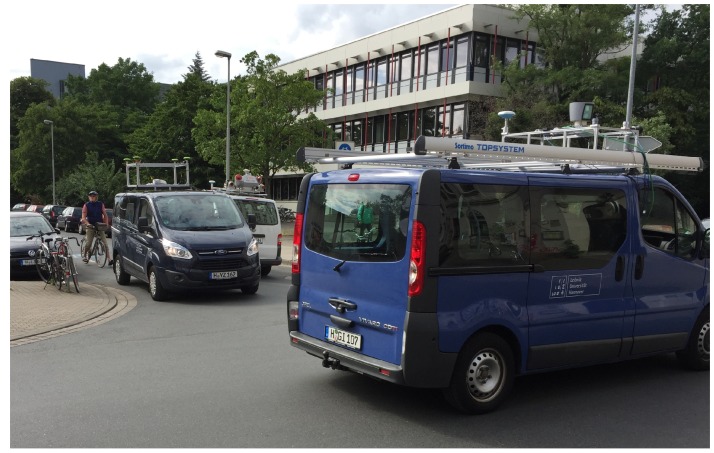
Photograph of a typical situation during the mapathon: in the first Meet & Greet scenario, all three cars meet at a junction (from left to right: IfE, IKG, GIH van).

Four scenarios with a duration of about 15 min each were designed in order to provide suitable data to evaluate the behavior of the dynamic sensor network. The characteristics of the selected urban area and the vehicle to vehicle visibility were the two main requirements taken into account in the design process. The following are the selected scenarios (Figure 5):

(a) Meet & Greet scenario in an urban area. In this scenario, the three vehicles navigate in an urban area, meeting each other at an intersection. In addition, a group of about 20 pedestrians takes part in the scenario, in order to provide data for the pedestrian detection and tracking research project (Figure 5a and Figure 6).
(b) Following scenario in an urban area. In this scenario, the three vehicles follow each other, starting in a relatively open area with low velocities, then, later on, speeding up on streets partially covered by vegetation (Figure 5b).
(c) Meet & Greet scenario in an area covered by vegetation. Here, Vehicles 1 and 2 follow each other in a residential area with low height buildings. The main constraint for the satellite visibility is the urban vegetation. Pedestrians are again part of the scenario and are observed for subsequent detection and tracking (Figure 5c).
(d) Mixed scenario. In this scenario, a combination of predefined trajectories with traffic rules of the area results in a scenario in which the three cars intermittently approach an intersection, combining Scenarios (a) and (c) above.

### 2.3. Experimental Results, Post-Processing and Storage

The experiment was carried out during the afternoon/evening on 12 June 2017 after the calibration of all mobile platforms had been done in the morning of the same day. All four aforementioned scenarios were run for about 15 min each. Between each pair of successive scenarios, breaks of about 45 min were required to properly finalize and backup the data from the previous experiment and to coordinate the following scenario. Weather conditions were fine and stable over the whole duration of the experiment; lighting conditions changed over time from sunny in the afternoon to dawn in the evening.

In total, each car spent about 90 min actively recording data in the context of the different scenarios while driving about 19 km on average. During this time, all three cars together recorded about 330,000 stereo image pairs, while the Velodyne laser scanner and the RIEGL mobile mapping system recorded 4.3 billion and 1.8 billion points, respectively.

**Figure 7:**
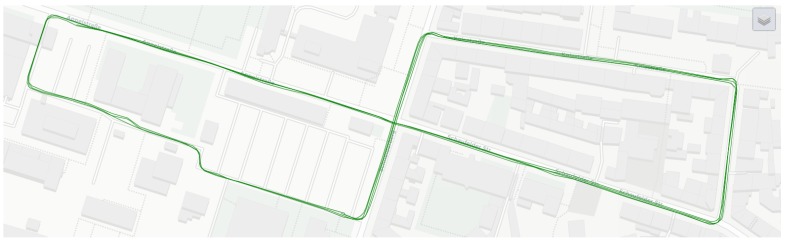
Reference trajectory for IfE van during the first Meet & Greet scenario as obtained by a JAVAD Sigma sensor.

In order to compute precise relative positions, a Leica AR25 GNSS antenna connected to different GNSS receivers permanently installed on the roof top of the Geodetic Institute was used as a reference station with a 10-Hz frequency. The NOVATEL GrafNav Software, as well as Terrapos REF were used after the measurements to perform GNSS Single Point Positioning (SPP), double difference DGPS and to derive tightly-coupled solutions. Figure 7 shows one reference trajectory.

The point cloud data from the RIEGL mobile mapping system were produced at different Levels Of Detail (LOD). LOD 0 included all original points, and LODs 1–3 were versions down-sampled by a factor of 4, 16 and 64, respectively. Due to the large data volume of LOD 0, this version of the point cloud was available in tiles of 15 m × 15 m size. In addition to the RIEGL solution, which included errors from drift in the internal GNSS system, a high-accuracy version of the point clouds was produced by a geometric adjustment of the point cloud including a correction of the vehicle trajectory [54].

After the experiments, in addition to the original recordings, records for all sensors were converted in a common spatial (ETRS89 (European Terrestrial Reference Frame 89)/UTM32N) and temporal frame (UNIX time extended to fractions of a second) in order to allow direct integration of measurements from different sensors. The data were then stored in a file server using non-proprietary ASCII formats, allowing direct import by most software packages.

All sensor recordings (original and derived versions) were associated with a metadata XML file containing relevant meta information about the origin of the data (e.g., sensor specifications, associated mobile platform, experiment/scenario IDs, spatial/temporal extent). The metadata schema was designed in accordance with the requirements of our own research; in addition to a number of mandatory non-sensor-specific metadata attributes, there were optional (e.g., sensor-specific) attributes, which were added only where necessary. For better access to the complex dataset, a metadata browser was developed, which traverses the data repository and summarizes all metadata in a spatial database. This database can then be filtered using the mandatory entries from the metadata schema, allowing access to data from specific experiments, from specific mobile platforms, from specific sensors or from a specific point in space and/or time.

**Figure 8:**
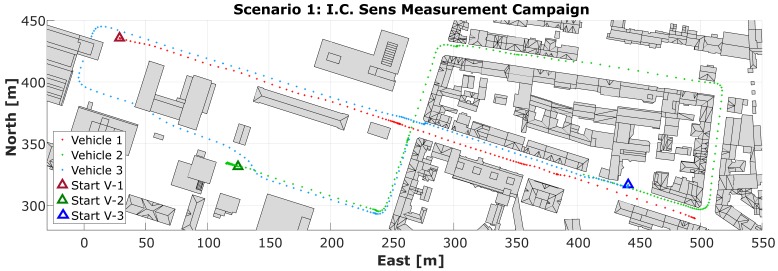
Schematic overview of the first Meet & Greet scenario, combining the measured GNSS data with a Level Of Detail 2 (LOD 2) city model as done in Section 3.3. i.c.sens, integrity and collaboration in dynamic sensor networks.

**Figure 9:**
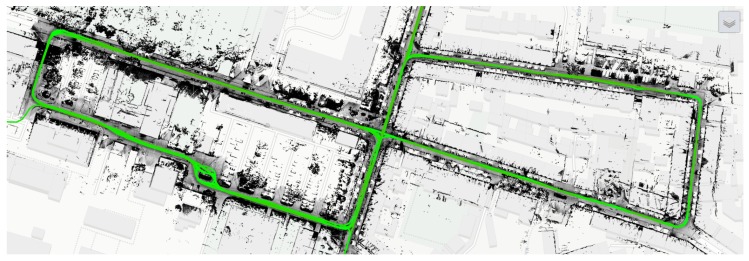
Example for the data preview on the i.c.sens website, showing a plot of the mobile mapping system point cloud from the same scenario, superimposed on a topographic base layer. Trajectories of the IKG van from all scenarios (green) are added as a separate layer. In a similar way, layers for all datasets can be combined to produce map mashups.

In order to allow previewing the contents of individual recordings without having to download large volumes of data, a visual preview was produced for each dataset, depending on the type of data either as vector data (e.g., downsampled trajectories for GNSS measurements) or as raster data, aggregating information from the raw data (e.g., rendered images from point clouds). All previews are available in a graphical user interface including a web map, where the data are shown in their spatial context (see Figure 8 and Figure 9). These tools allow a pre-selection of relevant data-sets using visual inspection and comparison. Further visual representations produced by the participants can be automatically included in this interface, allowing full visual-analytics capabilities.

## 3. Exemplary Results on Integrity and Collaboration

Within the research training group, in total, nine individual PhD projects are carried out focusing on aspects of integrity and collaboration in dynamic sensor networks. The following subsections present their main ideas and show the current results.

### 3.1. Inconsistency Measures for GPS-Derived Positioning

**Figure 10:**
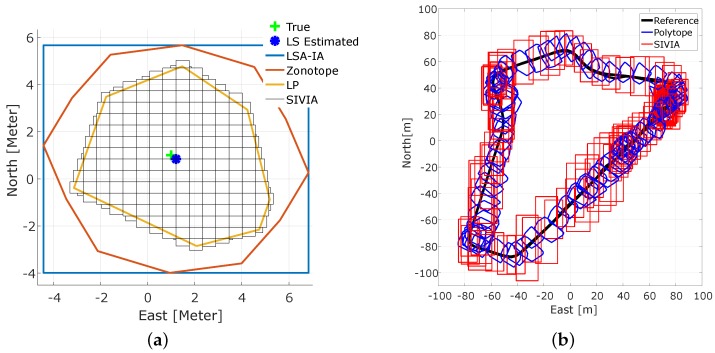
Comparison of different bounding methods: (**a**) 2D simulated solution sets of all methods, (**b**) Set Inversion Via Interval Analysis (SIVIA) and Linear Programming (LP) inconsistency zones from the scenario 3 test drive. LSA-IA, Least Squares Adjustment based on Interval Analysis.

We applied interval-based error bands to GPS pseudorange measurements. Interval-based approaches and set theory are alternatives to derive integrity measures that do not suffer from assumptions about probability distributions and that intrinsically have an error propagation different from quadratic variance propagation [55]. A theoretical study has been carried out to derive those interval error bands for different types of measurements and sensors [22,56]. Following that, four different deterministic bounding methods were applied to GPS positioning, namely: Least Squares Adjustment based on Interval Analysis (LSA-IA), an extension of LSA-IA by means of zonotopes [57], Linear Programming (LP) applied to the navigation equation linearized by Taylor expansion and, finally, solving the non-linear navigation equation by Set Inversion Via Interval Analysis (SIVIA); for the mathematical developments, see [58]. The main difference between the above-mentioned methods is that LSA-IA and zonotopes provide confidence zones similar to the well-known error ellipses from stochastic approaches, while LP and SIVIA provide inconsistency measure bounding zones. Figure 10a shows a simulated 2D example, where the SIVIA solution is the union hull of all small interval boxes, and Figure 10b shows polytopes (in blue) obtained from LP and the interval box (in red) obtained from SIVIA, both of them applied to the GPS code measurements with constant error bounds equal to 6 m. The difference between SIVIA solutions in Figure 10a,b is that in the first one, a bisection is performed inside the algorithm, while in the second, only iterative contraction of the initial interval box is performed, which is much faster and feasible for real-time applications.

**Figure 11:**
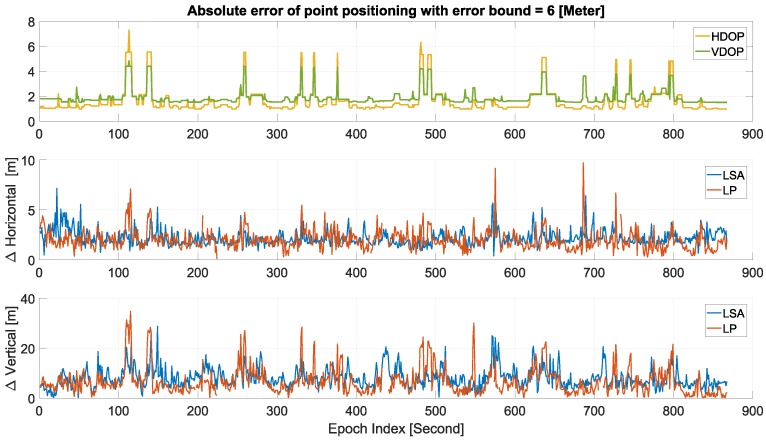
Point positioning errors in horizontal and vertical directions for the trajectory of Figure 10b. Top panel: DOPvalues, Middle panel: horizontal error in (m), Bottom panel: vertical position error in (m).

Figure 11 shows the absolute errors in the horizontal and vertical direction of the LSA and LP point positioning of the same test drive in Figure 10b. With bad geometry (large DOP values), the LP solution deteriorates and showed higher peaks than LS. The missing epochs in the LP solution were related to the empty set where outliers are detected, but not excluded. However, the overall performance of LP showed better results than LS. Detailed information of each method and results from simulated and real data (Mapathon Scenario 3) can be found in [58].

As a conclusion, LP point positioning was more accurate and precise than LS, but it was more sensitive to the navigation geometry. Moreover, the LP and SIVIA algorithms provided guaranteed bias detection and exclusion.

### 3.2. Development of a Filter Model with Integrity Measures

In addition to snapshot methods (see Section 3.1), filters are applied in kinematic positioning. Here, both the observations and the system knowledge are superimposed by random and set-membership uncertainties. For this purpose, the nonlinear observation and system equations of the filter are extended to the imprecise case and solved by methods considering both random and set-membership uncertainties. This includes the extension of the available basic concepts by elements of guaranteed parameter estimation, as well as filtering for merging the knowledge from observation and about the system. A particular focus of this project is on the propagation of imprecision, with the goal of significantly solving the problem of overestimation, which usually occurs with interval mathematics when finding suitable reformulations. The required interval matrix inversions may be addressed by approximations either leading to the loss of some solutions [59] or to some overestimation effects unless set inversion techniques are used [60].

In [61], a new filter model called the Set-membership Kalman Filter (SKF) was developed with both statistical uncertainty and Unknown, But Bounded (UBB) uncertainty, and the uncertainty propagation in the nonlinear dynamical system was analyzed. The main loop of this new algorithm included one prediction step and one correction step with measurement information, and the key part in each loop was to solve an optimization problem. The solution of the nonlinear programming problem produced the optimal estimation for the state, which was bounded by ellipsoids. For applications of SKF on real datasets, one trajectory estimation experiment of a multi-sensor system can be found in [62].

**Figure 12:**
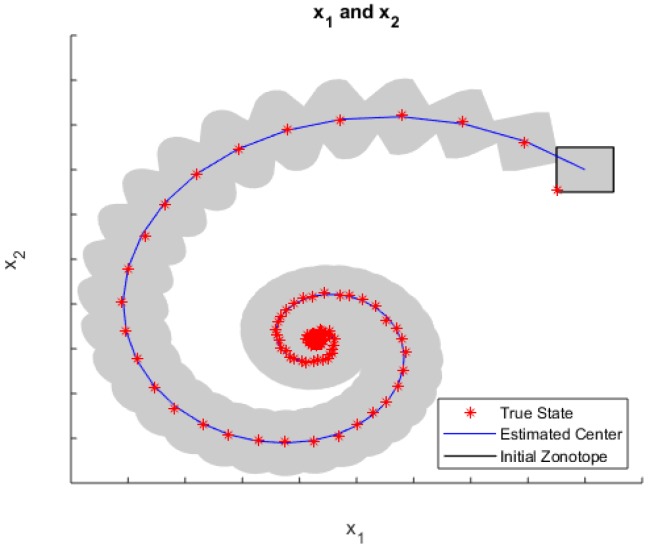
Qualitative evolution of the state estimation under the Zonotopic and Gaussian Kalman Filter (ZGKF) for a hybrid linear time-invariant system.

Another interesting set-based approach is using zonotopes to bound UBB uncertainty, then combining it with statistical uncertainty; see the Zonotopic and Gaussian Kalman Filter (ZGKF) in [63]. The ZGKF filter model can also be extended to hybrid Linear Time-Invariant (LTI) systems, which contained an Ordinary Differential Equation (ODE) and a discrete measurement equation. See the following Figure 12 for a simulated example of ZGKF on a hybrid system.

In this example, the five-dimensional state vectors in a hybrid LTI system were estimated using ZGKF, and the projected results on $x_{1}$ and $x_{2}$ are given above. In each epoch, the grey area demonstrated a projected zonotope, which contained all expectations of possible Gaussian distributions. There were uncountably infinite normal distributions after the estimation since the set-membership uncertainties were introduced into the dynamical system at the beginning. The blue line connects all the zonotope centers, and the red stars are the projected true states.

### 3.3. Integrity Information-Based Georeferencing

External geometric information like knowledge about parallel or perpendicular walls can be used to improve the integrity of georeferencing. In fact, numerous possibilities exist for providing pose information of a kinematic Multi-Sensor System (MSS) in its environment. However, these differ in terms of feasible degrees of freedom, position accuracy, geometrical long-term stability and range [64]. Mainly due to the absence of accurate and reliable GNSS observations, georeferencing with integrity aspects of a kinematic MSS within complex indoor (e.g., office spaces) and inner-city outdoor environments (e.g., with many high buildings and vegetation) is very challenging and expensive, primarily due to shadowing and multipath effects. Thus, demands for real-time processing or high accuracy are difficult to achieve.

**Figure 13:**
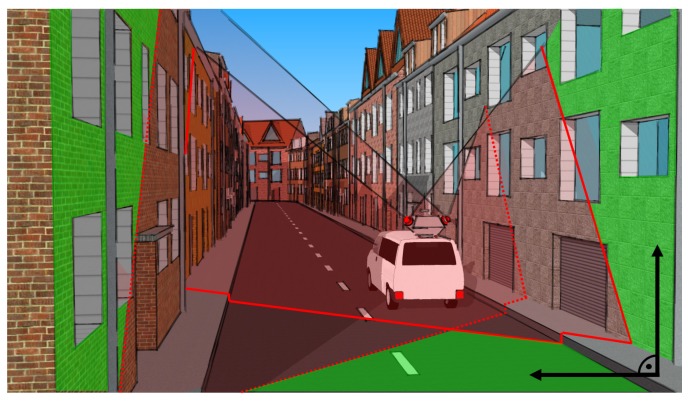
Exemplary illustration of a scenario for information-based georeferencing of a kinematic MSS (white vehicle) with classified building facades and road surface (green), as well as their geometric relation (black).

In order to ensure accurate and reliable pose information of a kinematic MSS, we combined point cloud information from a laser scanner with independent geometric conditions of the direct environment; see Figure 13. We defined geometric equality constraints (e.g., concurrency and perpendicularity in relation to surfaces like walls, facades and ceilings) and took them into account during pose estimation in an Iterated Extended Kalman Filter (IEKF), which is applicable to implicit measurement equations. We extended this approach by the mentioned nonlinear equality constraints and full error propagation of the filtered states [65]. Results from simulated scenarios, as well as from real indoor experiments showed significant improvements (Root Mean Square Error (RMSE) of 6 mm compared to 400 mm when not considering such geometrical constraints for pose estimation).

Furthermore, the approach will be extended to nonlinear inequality constraints in order to take information about the building construction process in terms of total tolerances into account and to consider robustness against outliers.

### 3.4. Interval-Based Simultaneous Localization and Mapping with Spatio-Temporal Uncertainties

When fusing information from multiple sensors, not only the assumptions about single sensor errors have to be taken into account, but also inter-sensor properties such as time synchronization or spatial calibration have to be considered. Existing approaches [27] often neglect these spatio-temporal parameters or assume them to be perfectly known. An example is the temporal offset between two sensor clocks that are not synchronized. We model the offset between two sensor clocks using intervals since the result is naturally bounded (e.g., accurate to ±10 ms ). Similarly, the transformation between two sensors is naturally bounded (e.g., accurate to ±1 cm).

To evaluate our approach, we used data from our measurement campaign, since it included all required sensors and the ground truth was provided. Additionally, we used data from the calibration process (see Section 2.1) that preceded our campaign to reliably compute bounds for the spatio-temporal calibration parameters. The description of a first probabilistic approach to compute the timestamp offset between a rotating laser scanner, its motor and a camera can be found in [66,67].

**Figure 14:**
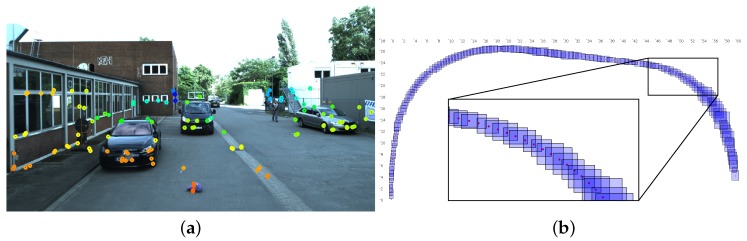
Results from the interval-based SLAM approach. (**a**) Image features color-coded by depth from close (red) via orange, yellow and green to distant (blue); (**b**) 2D position boxes (blue) that contain the vehicle’s true position (red).

The preliminary results of our interval-based SLAM approach are depicted in Figure 14. Image features were extracted and augmented with depth information by a laser scanner (cf. Figure 14a). Afterwards, a position box was computed that was guaranteed to contain the robot’s true position (cf. Figure 14b). In between, we used GPS measurements to contract the position boxes to keep them from growing indefinitely due to drift.

### 3.5. Collaborative Acquisition of Predictive Maps

Integrity information or high-precision and up-to-date models of the environment are mandatory in order to operate self-driving cars safely. Natural environments contain many dynamic objects and change over time. Since a permanent observation of very large areas is impossible, it is very challenging to keep such a model up to date. However, there would always be a first time visit to an area that has changed.

The environment can change in different ways, e.g., abruptly, gradually or periodically. Objects can have a different lifetime or stability. They can be permanent, like a building, or temporal, like a parked vehicle. Some objects are slightly moved on a regular basis, like recycling bins, or they change their appearance, like open and closed doors. Vegetation grows or changes, according to seasons, or can be removed or cut down by human intervention. Knowing the temporal behavior of different objects or areas in the environment improves the reliability of a map and can help to predict their future state. Being able to predict the map state at a certain point in time allows us to reduce the discrepancy between the expected and the actual (observed) state. Thus, an observer using the map has to deal with a smaller amount of unexpected measurements, or outliers.

We aim to create an updatable and extendable long-term map that takes into account the dynamics of an urban environment. Changes can be classified according to the duration of their stability, e.g., days, weeks, months and years. Moreover, a confidence score that represents the likelihood of an object or area in the map to be static (for a defined period) can be derived.

**Figure 15:**
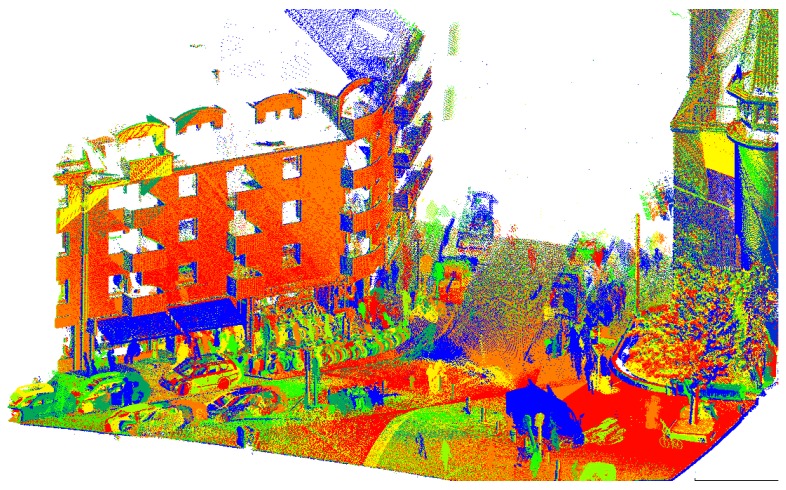
Point cloud from seven measurement campaigns. Points are colored by campaign (blue = oldest, red = most recent).

In addition to the mapathon described above, we ran extra measurements on a biweekly basis to obtain continuous data for an overall duration of one year. We used the Riegl VMX-250 Mobile Mapping System to record dense LiDAR data on a fixed 20-km route in the city of Hannover. The route partly overlaps with the mapathon routes and covers different typical urban areas, e.g., inner city streets, high buildings, parking lots, residential areas, tramlines, controlled and uncontrolled intersections and construction sites. In total, we had 25 measurement campaigns, which took place from March 2017–March 2018. An example scene of the resulting LiDAR data is shown in Figure 15.

Some preliminary results have been presented in [68], based on LiDAR data from earlier measurement campaigns in Hannover-Badenstedt. There, we analyzed the temporal behavior of typical objects in an urban scene and distinguished between static and dynamic objects by a simple threshold. Therefore, the aligned point clouds of all measurements were segmented and accumulated into a voxel grid. The next step was an analysis of typical urban objects. The occupancy state of all voxels of an object was examined for all measurements. As a result, an object was rated to be static and permanent, if at least 25% of its voxels were occupied in at least 70% of the measurements. In the future, we expect to obtain much better results with our new, biweekly measurement campaigns, since it is not only more data, but also the capture dates of the campaigns were distributed evenly in time.

### 3.6. Quality Measures for 3D Semantic Segmentation Using Deep Learning

By exploiting the geometric correspondence between camera pixel and 3D points, we created a dataset for 3D point cloud classification. We first annotated the images taken during the mapathon using pre-trained CNN (convolutional neural network) models for semantic segmentation. To that end, we ran PSPNet, which was trained on the cityscapes dataset [69]. Then, using the known pose of all cameras, we transferred the labels to the 3D points. The dataset constructed in this way contained about one billion labeled points.

However, the dataset contained label noise, mainly because of calibration and classification errors and differences in capture time and location between the sensors, both leading to occlusions. We estimated the similarity, measured as the mean Intersection over Union (IoU) between projected labels and the ground truth, to be approximately 32%, based on an evaluation using hand-labeled reference data. We currently are developing machine learning techniques to investigate and improve the quality of our dataset. The final result should be a classifier that is able to perform semantic segmentation in point clouds using only a few (or even none) manually-labeled 3D points.

**Figure 16:**
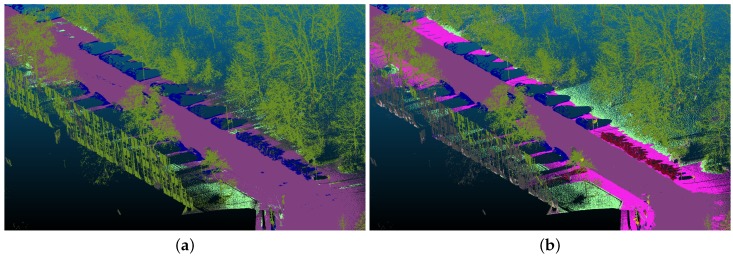
Semantic segmentation of 3D point clouds, using labels transferred from image segmentation, without (**a**) and with (**b**) estimated label noise.

Our current result (see Figure 16) showed that it was possible to do semantic segmentation using this approach. Furthermore, we were able to learn the noise and errors that were induced by label transfer. As shown in Figure 16b, we estimated and removed mislabeled 3D points and improved the IoU to approximately 54%. In our future research, we will try to reduce the amount of label noise to a level that is similar to the current state-of-the-art for semantic segmentation in images.

### 3.7. Optimal Collaborative Positioning

Collaborative positioning is in our perspective a promising technique, in which a group of dynamic nodes (pedestrians, vehicles, etc.) equipped with different (time synchronized) sensors can increase the quality of each individual position, navigation and timing information by sharing and exchanging information, as well as by performing measurements between nodes or to elements of the environment (urban furniture, buildings, etc.).

In order to get insight into the behavior of such dynamic networks and to evaluate the benefits of collaboration with respect to single vehicle approaches, a realistic simulation tool for collaborative navigation systems was developed. Satellite navigation, inertial navigation, laser scanner and photogrammetric techniques were combined, providing not only an algorithm capable of fusing different sensor measurements with localization or positioning purposes, but also the possibility to evaluate the correlations and dependencies of estimated parameters and observations.

**Figure 17:**
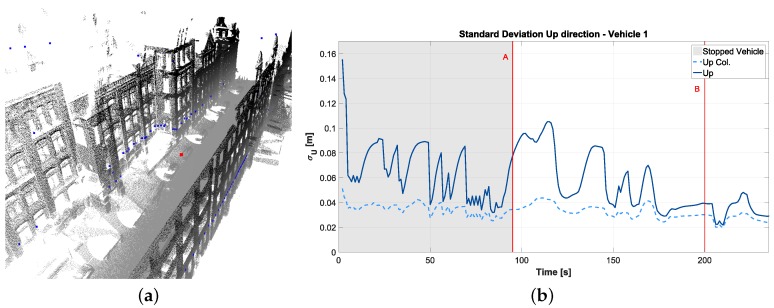
(**a**) Environmental model obtained from preprocessed point cloud. (**b**) Standard deviation results from the LKF estimation in the up direction (collaborative approach versus single vehicle).

In a first step, a batch algorithm based on least-squares estimation using an environmental model obtained by digitalization of elements of the environment [70] was implemented. In order to include the system dynamics, a linearized Kalman filter fusion algorithm [71] was set up [72]. In addition, we improved the environmental model to contain either a 3D city model or preprocessed laser scanner point clouds, in this way obtaining a more realistic representation of the environment; cf. Section 3.5. In Figure 17a, the representation of the preprocessed laser scanner point cloud (black dots) is presented, together with the vehicle position (red dot) and the obtained simulated V2I intersections (blue dots). These points are used as discrete time equations in the correction step of our linearized Kalman filter.

These results obtained in both approaches showed the very high influence of the network geometry on the state estimation and in general an improvement in the precision and accuracy of the state estimation using the collaborative approach with respect to the single vehicle approach. The standard deviation values selected for the white noise measurement simulation constrained the values for the accuracy and precision. If the standard deviation value for the laser scanner V2I measurements were selected to be 10 cm (lateral and longitudinal), the RMSE and estimated standard deviation of the positioning estimation would be approximately 1 cm. The amount of improvement of the collaborative approach with respect to the single vehicle approach depended on the configuration used and on the standard deviation values assumed for each measurement (reaching in a 3D case values in the range of 40% improvement, cf. Figure 17).

### 3.8. Dynamic Control Information for the Relative Positioning of Nodes in a Sensor Network

One important input measurement in collaborative positioning is the relative positioning information between the sensor nodes. Enabling the communication and transmission of relative poses between the vehicles allows one to incorporate them as dynamic control information to enhance the positioning. This leads to the need for techniques for precise 3D object reconstruction to derive the poses of other vehicles relative to the position of the observing vehicle. In this context, stereo cameras provide a cost-effective solution for sensing a vehicle’s surroundings. Most of the existing techniques for vehicle detection and pose estimation have been restricted to a coarse estimation of the viewpoint in 2D (e.g., [73,74]), whereas the precise determination of vehicle pose, especially of the orientation, and vehicle shape is still an open problem that has been addressed here.

**Figure 18:**
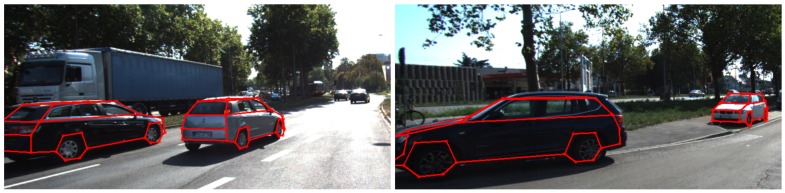
Estimated vehicle models backprojected to the image. (**Left**): models assessed to moving cars; (**Right**): models assessed to parked cars.

A first approach for the detection and modeling of vehicles from stereo images was presented in [75]. Here, a generic 3D object detection approach was combined with a state-of-the-art image object detector for the detection of vehicles, and a deformable vehicle model was estimated for every vehicle detection based on a set of reconstructed 3D points. A more mature approach for the precise model-based reconstruction of vehicles can be found in [76], where the model fitting procedure was extended by incorporating automatically-derived scene knowledge and further information from the images. With a detection score of up to 94%, correct pose estimations for up to 82% of the detected vehicles and a mean orientation error of less than 4${}^{\circ }$, we achieved promising results for the generation of V2V observations. Figure 18 shows exemplary results of the estimated model wireframes backprojected to the the image plane.

### 3.9. Collaborative Pedestrian Tracking

**Figure 19:**
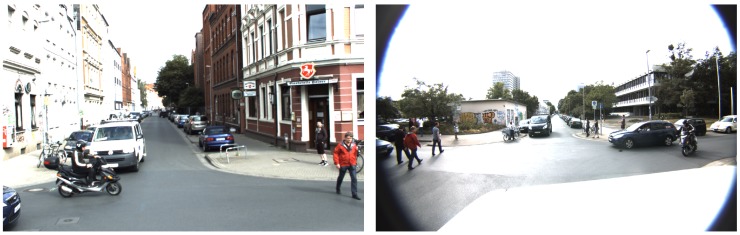
Pedestrians are observed from two cameras in the mapathon.

People detection and tracking is one of the most vigorous research topics in computer vision and photogrammetry. Today, pedestrians can be localized and traced automatically in image sequences, e.g., [77]. However, applying the detection and tracking results for practical applications still requires a significant improvement of the quality of the generated trajectories. The focus of this project was to develop a pedestrian detection and tracking system based on tracking-by-detection with high reliability and accuracy. To do that, we detected and tracked multiple persons from different perspectives using multiple moving stereo cameras. A scenario was set up in which multiple moving cars collaboratively carried out the following task (see Figure 19): persons or groups of persons, typically walking on pavement of a street or crossing a street at a zebra-crossing, were detected from stereo image pairs and tracked as long as the persons were present in the captured images. This information was then broadcast to nearby cars. The broadcasting to car-nodes in the network was supposed to happen asynchronously; thus, every stereo system started its data capture and processing independently of the other sensors. If results from a nearby sensor were available, they would be fused with the information available locally. Missing information from a certain viewpoint could thus be filled in by the others. This is an advantage that tracking in a single perspective cannot provide. Using that additional information, the robustness against occlusion and the reliability of tracked trajectories could be improved.

Our developed tracking system was evaluated using the mapathon data, in which stereo images capturing pedestrian movements from up to three different viewpoints employing three car-nodes were generated.

In the first attempt to detect pedestrians in each image of a sequence, we developed a regions proposal generator to guide a classifier where to examine image regions that may contain pedestrians; see [78]. We presented a proposal framework employing both 3D information derived from stereo images and RGB cues to generate pedestrian bounding box proposals with approximately 90% recall with 2000 region proposals. Currently, we are extending and improving our system to include classification of the detected region proposals using sophisticated and accurate classifiers like convolutional neural networks.

## 4. Discussion and Conclusions

Integrity and collaboration will play a major role in future positioning and navigation applications, especially when heading towards autonomous driving in harsh GNSS environments. Sharing data from multi-sensor platforms enables new applications and yields benefits in accuracy, integrity and continuity for all participants.

In this paper, we presented approaches based on interval mathematics and set theory in order to provide alternative and/or additional integrity measures to the classical, purely stochastic approaches. This includes the derivation of inconsistency regions for positional states, extended filters to incorporate both random and bounded uncertainty components, as well as additional knowledge on the geometric relations between objects. Furthermore, uncertainty about time synchronization of and between multi-sensor platforms can be described in a natural way by intervals yielding bounding regions for the estimated states. The dedicated analysis of scan drives repeated over one year enables classifying the variation rate of our continuously changing environment and thus associating trustworthiness information to enhanced future maps.

Collaborative scenarios are often ad-hoc situations. However, an optimal selection and attribution of sensors to different nodes, as well as a reliable identification and relative tracking of all participants will further strengthen these dynamic networks. The design and data acquisition of dedicated test drives was described in detail. This included three multi-sensor vehicles, as well as pedestrian choreography to create such dynamic networks. Results with regard to the pedestrian tracking approach and the identification of other vehicles and their relative pose estimation by means of stereo images were discussed. Finally, a collaborative positioning simulator was developed including environmental models and multiple vehicles equipped with laser scanners, cameras, GPS, IMU and odometers. The simulation results showed the clear benefits of collaboration and enabled studying the contribution of each measurement to the state estimation of all vehicles.

## Figures and Tables

**Table 1:** Camera setups. IfE, Institut für Erdmessung; GIH, Geodetic Institute Hannover; IKG, Institute for Cartography and Geoinformatics.

	IfE Van	GIH Van	IKG Van
Cameras	Allied Vision	Pointgrey	Allied Vision
	AV MAKO G-234C	GS3-U3-23S6C-C	AV MAKO G-234C
Lenses	Fujinon	Tamron	Schneider Kreuznach
	CF12.5HA-1	M111FM08	Cinegon 1.8/4.8-0902
Focal length	12.5 mm	8.0 mm	5.0 mm
Image size	1936 × 1216	1920 × 1200	1936 × 1216
Pixel size	5.86 μm	5.86 μm	5.86 μm
Frame rate	25 fps	25 fps	25 fps
Base length	0.91 m	0.93 m	0.85 m
